# Development of Polioencephalomyelitis in Cesarean-Derived Colostrum-Deprived Pigs Following Experimental Inoculation with Either *Teschovirus A* Serotype 2 or Serotype 11

**DOI:** 10.3390/v9070179

**Published:** 2017-07-08

**Authors:** Franco Matias Ferreyra, Bailey Arruda, Gregory Stevenson, Kent Schwartz, Darin Madson, Kyoung-Jin Yoon, Jianqiang Zhang, Pablo Piñeyro, Qi Chen, Paulo Arruda

**Affiliations:** Veterinary Diagnostic Laboratory, Department of Veterinary Diagnostic and Production Animal Medicine, Iowa State University, 1850 Christensen Drive, Ames, IA 50011-1134, USA; francomf@iastate.edu (F.M.F.); wilberts@iastate.edu (B.A.); stevengw@iastate.edu (G.S.); kschwart@iastate.edu (K.S.); madson@iastate.edu (D.M.); kyoon@iastate.edu (K.-J.Y.); jqzhang@iastate.edu (J.Z.); pablop@iastate.edu (P.P.); chenqi@iastate.edu (Q.C.)

**Keywords:** Teschovirus encephalomyelitis, *Teschovirus A*, *Porcine teschovirus*, Porcine teschovirus 2, Porcine teschovirus 11, poliomyelitis, Teschen disease, Talfan disease

## Abstract

Teschovirus encephalomyelitis is a sporadic disease associated with *Teschovirus A* (PTV) serotype 1 and, less frequently, other serotypes. In recent years, the number of cases submitted to the Iowa State University Veterinary Diagnostic Laboratory with a history of posterior paresis has increased. Submission histories from various regions of the United States suggest a trend for clinical disease to persist in herds and affect a wider age-range of pigs than historically reported. Polioencephalitis and/or myelitis was consistently present and PTV was detected in affected neural tissue by PCR in a portion of cases. Sequencing from two clinical cases identified PTV-2 and PTV-11. To assess neuropathogenicity of these isolates, 5-week-old cesarean derived and colostrum-deprived pigs were assigned to three groups: negative control (*n* = 4), PTV-2-inoculated (*n* = 7), and PTV-11-inoculated (*n* = 7). Three PTV-2-inoculated pigs developed mild incoordination of the hind limbs, one of which progressed to posterior ataxia. While all PTV-11-inoculated pigs showed severe neurological signs consistent with Teschovirus encephalomyelitis, no evidences of neurological signs were observed in sham-inoculated animals. All PTV-2- and PTV-11-inoculated pigs had microscopic lesions consistent with Teschovirus encephalomyelitis. To our knowledge, this is the first description of PTV-11 and experimental study demonstrating the neuropathogenicity of PTV-11 in the United States.

## 1. Introduction

Teschovirus encephalomyelitis (TE), previously Teschen or Talfan disease, is a neurologic condition of pigs commonly characterized by locomotor disturbances including ataxia, paresis, and/or paralysis [1]. Teschen disease (also known as Klobouk’s disease) was first recognized in the Czech Republic in 1929 as a fatal encephalomyelitis of pigs caused by a highly pathogenic strain of *Teschovirus A* (formerly named *Porcine enterovirus* and then *Porcine teschovirus*) [2]. *Teschovirus A* consists of 13 (sero)types, *porcine teschovirus* (PTV) 1 to 13, and is a single-stranded, linear, non-segmented RNA virus of the genus *Teschovirus*, family *Picornaviridae* [3]. To date, outbreaks of mild disease have been described in the United States [4,5,6,7], although the most severe form of TE caused by highly virulent strains of PTV-1 has not been reported. However, outbreaks of TE due to PTV-1 have been recently reported in Haiti, Dominican Republic, and Canada [8,9,10].

Historically, PTVs were classified within the genus *Enterovirus* based on viral morphology, cytopathic effect (CPE) produced, serological assays, and replication in different cell lines [7,11,12]. Recently, PTVs have been reclassified by nucleotide sequence matching, genomic organization, and phylogenetic analysis [13,14,15]. *Porcine enterovirus* (PEV)-1 to -7 have been renamed PTV-1 to -7 and PEV-11 to -13 were renamed PTV-8 to -10. PTV-11 to -13 are the (sero)types most recently identified [16,17,18]. Despite the reported high prevalence of PTV in feces, clinical disease is observed sporadically [19,20,21,22]. Although multiple serotypes have been identified and genetically characterized, the neuropathogenicity of some of these serotypes has not been fully elucidated. The aim of this study was to determine the neuropathogenicity of the PTV-2 and PTV-11 isolates.

## 2. Materials and Methods

### 2.1. Virus Inoculum

Strains PTV-2 USA/IA65463/2014 and PTV-11 USA/IA09592/2013 were isolated at the Iowa State University Veterinary Diagnostic Lab (ISU VDL) from samples of central nervous system (CNS) tissue from pigs with neurologic disease. These viruses were first identified using a previously described nested polymerase chain reaction (PCR) [14] and further characterized by virus isolation and molecular sequencing of the VP1 capsid protein. For experimental inoculation PTV-2 passage 8 and PTV-11 passage 7 were grown in the porcine kidney 15 (PK-15; ATCC CCL-33) cell line. Nucleotide sequence data for both isolates is available in GenBank under accession numbers KY594021 (PTV-2 USA/IA65463/2014) and KY594022 (PTV-11 USA/IA09592/2013).

### 2.2. Next Generation Sequencing and Phylogenetic Analysis

Complete genome sequences of PTV-2 USA/IA65463/2014 and PTV-11 USA/IA09592/2013 isolates were determined by next generation sequencing on the MiSeq platform (Illumina, San Diego, CA, USA) following previously established procedures [23]. Total DNA/RNA was extracted from virus cell culture and purified with MagMAX viral RNA isolation kit (Life Technologies, Carlsbad, CA, USA) and a Kingfisher 96 instrument (Thermo Scientific, Waltham, MA, USA) [24]. DNA was then removed with RNase-Free DNase Set (Qiagen, Valencia, CA, USA) from the total DNA/RNA, and the remaining RNA was purified with an Agencourt RNAClean XP (Beckman Coulter, Indianapolis, IN, USA) kit according to the manufacturer’s directions. Purified RNAs were fragmented to approximately 250 bp and reverse transcribed to double stranded DNAs with NEXTflex Rapid RNA-Seq Kit. DNA library was constructed with Nextera XT DNA library preparation kit (Illumina, San Diego, CA, USA). Sequencing was performed on MiSeq with 300-cycle MiSeq Reagent Micro Kit v2 (Illumina, San Diego, CA, USA) to generate paired-end 2 × 150 bp reads for each sample. PTV sequences were extracted from raw sequencing output with BWA-MEM (v 0.7.15) [25] as previously described with modification [26]. Specifically, the reference genome library in the analysis pipeline was built by searching against NCBI nucleotide database with query ‘(complete genome) AND “Teschovirus” (porgn: txid118139)’. Extracted PTV fragments were assembled with ABySS (v 1.5.2) [27] and SeqMan Pro version 11.2.1 (DNASTAR, Inc., Madison, WI, USA) as previously described [24]. For phylogenetic analysis, both PTV-2 USA/IA65463/2014 and PTV-11 USA/IA09592/13 were aligned with 79 PTV polyprotein sequences obtained from GenBank. Multiple sequence alignment and sequence comparisons were carried out using the ClustalW algorithm by Geneious R9 software (Biomatters Ltd, Auckland, NZ). Phylogenetic trees were reconstructed with the Neighbor Joining (NJ) method using the Tamura–Nei model by Geneious R9 software. The confidence of the internal branches was evaluated performing 100 bootstrap replications.

### 2.3. Animals

Eighteen cesarean-derived colostrum-deprived pigs (CDCD) were purchased from a commercial source. Fecal swabs were collected 5 days prior to inoculation and tested using a nested PCR targeting PTV, *Sapelovirus A*, and *Enterovirus G* [14]. All samples were negative. All procedures were approved by the Institutional Animal Care and Use Committee of Iowa State University (Log Number: 6-15-8040-S, approval date: 14 January 2016).

### 2.4. Experimental Design and Clinical Evaluation

Pigs were randomly assigned to three treatment groups: negative control (*n* = 4); PTV-2-inoculated (*n* = 7); and PTV-11-inoculated (*n* = 7). Each group was housed in separate rooms and fed ad libitum with commercial feed. Pigs were sedated by intramuscular injection of Telazol 500 mg (Zoetis, Kalamazoo, MI, USA), Ketamine 250 mg (VET one, Boise, ID, USA), Xylazine 250 mg (VET one) at 4.4 mg/kg prior to inoculation. The inoculum was administrated intravenously using a 25-gauge butterfly catheter (Terumo Surflo Terumo Corporation; Shibuya, Tokyo, Japan) ensuring the delivery of virus. Negative control animals were inoculated with 3 mL of Eagle’s minimum essential medium (MEM 11095, Life Technologies); animals in PTV-2- and PTV-11-inoculated groups were inoculated with 3 mL of 10^6^ TCID50/mL of PTV-2 USA/IA65463/2014 or PTV-11 USA/IA09592/2013 (Table 1). Animals were evaluated every 48 h for the presence of clinical signs (Table 2). Serum samples were collected on day post-inoculation (DPI) 0, 11, and 21.

### 2.5. Necropsy

Eight pigs were necropsied at DPI 11 (negative control: *n* = 2; PTV-2-inoculated: *n* = 2; PTV-11-inoculated: *n* = 4). At DPI 16 a PTV-2-inoculated pig was necropsied due to clinical signs. The remaining piglets were necropsied at DPI 21. Prior to euthanasia a video of each animal was taken. A set of tissues including cerebrum; cerebellum; brainstem; spinal ganglion; sciatic nerve; and cervical, thoracic, and lumbar sections of spinal cord were fixed in 10% formalin for histopathologic examination.

### 2.6. Histopathologic Examination

All nervous system tissues were processed for routine histopathologic evaluation. Briefly, after 48 h fixation on 10% buffered neutral formalin, tissue samples were embedded in paraffin blocks, cut on 4 μm sections, and stained with hematoxylin and eosin. All sections were scored double blinded by two veterinary pathologists (P.A. and B.A.). Lesions were scored based on foci of gliosis and cellular infiltration of Virchow–Robins spaces (Table 3).

### 2.7. Serum Neutralization Test

Antibody neutralizing activity was evaluated at 0, 11, and 21 DPI. Results were corroborated by performing an indirect immunofluorescence assay (IFA). Briefly, all sera samples were inactivated at 56 °C for 30 min and aliquots of 75 μL were two-fold diluted in Dulbecco’s Modified Eagle Medium (DMEM 11995, Life Technologies) supplemented with 5% fetal bovine serum, 100 IU/mL penicillin, 100 μg/mL streptomycin, 50 μg/mL gentamicin, and 0.25 μg/mL amphotericin B. Equal volumes of diluted serum and PTV-2 and PTV-11 at 150 TCID_50_/75 μL were incubated for 1 h at 37 °C in a 5% CO_2_ humidified atmosphere. Afterward, 100 μL of the serum–virus mixture was added to microplate wells containing pre-confluent PK-15 monolayers and incubated at 37 °C. Cell controls were set up by adding 100 μL of DMEM to monolayers. Virus controls were made by adding residual incubated virus stock to monolayers. After 3 days, monolayers were assessed daily for the presence of CPE. On the fifth day, microplate wells were fixed with a solution of 80% ethanol at 4 °C. Infected cells were incubated with 50 μL of Porcine teschovirus/enterovirus antiserum (reagent code: 362-PDV, National Veterinary Service Laboratory, Ames, IA, USA) for 1 h at 37 °C, followed by 3 washes with PBS-T and revealed by incubation with 50 μL of fluorescein isothiocyanate (FITC) labeled anti-swine IgG (γ) (KPL Inc. Gaithersburg, Maryland, USA) and incubated for 1 h at 37 °C. The presence of positive infected cells was confirmed by fluorescence microscopy (OLYMPUS IX71; Olympus Corporation, Tokyo, Japan). The reciprocal of the highest serum dilution resulting in >90% reduction of staining as compared to the negative serum control was defined as the virus neutralization (VN) titer of the serum. A VN titer of ≥8 was considered positive.

### 2.8. Real-Time Reverse Transcription Polymerase Chain Reaction

For the extraction of nucleic acids from CNS tissues, pooled sections of brainstem, cerebellum, cerebrum, and spinal cord from each animal were homogenized with Earl’s minimum media. Feces were collected at the time of necropsy and immersed in 1 mL of PBS. Both samples were stored at −80 °C until further processing. Briefly, nucleic acids were extracted from aliquots of 50 μL of sample using a MagMAX96 Total RNA Isolation Kit (Applied Biosystems, Life Technologies) and a KingFisher Flex Purification System (Thermo Fisher Scientific, Pittsburgh, PA, US) following the instructions of the manufacturer. Primer and probe combinations for 5′ Taq nuclease assay using fluorescent 3′ minor groove binding DNA probe were designed targeting the 5’ untraslated region (UTR). Primer and probe sequencings are: PTV-forward GGTGGCGACAGRGTACAGA, PTV-reverse CCTGCATTCCCRTACAGGAACT and PTV-probe 6-carboxyfluorescein (FAM)-TGCRTTGCATATCCCTAG-MGB-BHQ. Amplification was carried out with a commercial quantitative reverse transcription PCR (RT-qPCR) kit (QuantiTect Virus + ROX Vial Kit, Qiagen), according to the manufacturer's instructions. The final protocol consisted of the addition of 5 μL of isolated RNA to 20 μL of RT-PCR mix (5 μL of 5× QuantiTect Virus Master Mix, 0.25 μL of QuantiTect Virus RT Mix, 1.0 μL XENO LIZ internal control reagent (Life Technologies), plus primers, to a final concentration of 0.4 μm; fluorogenic TaqMan MGB probe, to a final concentration of 0.2 μm; and RNase-free water up to 20 μL), and then the tubes were subjected to a first RT step at 50 °C for 20 min, followed by 5 min at 95 °C and 40 cycles of 15 s at 95 °C and 1 min at 60 °C.

### 2.9. Virus Re-Isolation

For all animals, aliquots of CNS tissue homogenate used for PCR were centrifuged at 800 *g* for 10 min at 4 °C and supernatants were filtered using 0.22 μm filters (Millex 638003 EMD Millipore Corporation, Billerica, MA, USA). A 100 μL aliquot was inoculated onto individual wells containing pre-confluent PK-15 monolayers and incubated for 1 h at 37 °C. After this step, the initial aliquots were discarded and culture plates were replenished with 100 μL of DMEM and incubated at 37 °C for 5 days and were examined daily for CPE. On the fifth day after a freeze-thaw cycle, the media from each well was collected separately and divided into two fractions of 50 μL each. From these, one fraction was used to reinoculate culture plate wells containing pre-confluent PK-15 monolayers and the other fraction was subjected to PCR. The re-inoculated culture plates were incubated for 5 days and CPE was examined daily. After this, culture plates were subjected to immunofluorescence staining to corroborate the re-isolation as described in the serology section.

## 3. Results

### 3.1. Next Generation Sequencing and Phylogenetic Analysis

**Figure 1:**
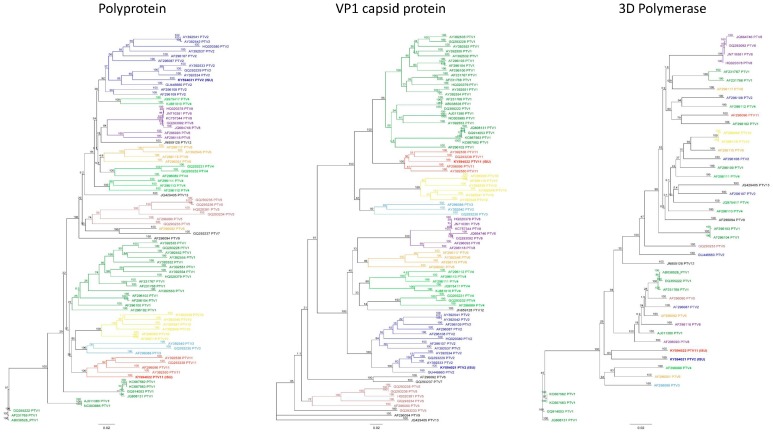
Phylogenetic trees illustrating the genetic relation of 79 unique polyprotein sequences with isolates PTV-2 USA/IA65463/2014 and PTV-11 USA/IA09592/2013. Polyprotein phylogenetic tree (**A**); Major capsid protein (VP1) gene phylogenetic tree (**B**); 3D^pol^ phylogenetic tree illustrating the genetic relation of 43 unique 3D gene sequences with isolates PTV-2 USA/IA65463/2014 and PTV-11 USA/IA09592/2013 (**C**). Branch distances expressed as percent of homology. Scale bar represents substitutions per site.

The genomic sequences of USA/IA65463/2014 isolate and USA/IA09592/2013 were determined using next generation sequencing technology. Phylogenetic analysis of the amino acid sequences encoding the polyprotein of PTV-2 and PTV-11 isolates identified in this study together with other PTV-1 to -13 sequences were conducted. Based on the VP1 nucleotide sequence, PTV-2 USA/IA65463/2014 clustered with other PTV-2 isolates and PTV-11 USA/IA09592/2013 clustered with others PTV-11 isolates. Pairwise comparison of the nucleotide sequence of the polyprotein revealed that the PTV-2 USA/IA65463/2014 had 82.5–89.25% nucleotide identity with 12 other PTV-2 isolates while the PTV-11 USA/IA09592/2013 had 85.8–87.2% nucleotide identity with other 4 PTV-11 isolates (Figure 1A). Additional phylogenetic analysis of the VP1 coding region also corroborated that these two isolates cluster with other members of the PTV-2 and PTV-11 serotypes (Figure 1B). Phylogenetic analysis of the 3D^pol^ gene indicates that both isolates share a high nucleotide homology for this coding region.

### 3.2. Clinical Evaluation

No clinical signs were observed in control animals, and all pigs remained cognitively aware throughout the study. A single pig in the PTV-2-inoculated group developed posterior ataxia at DPI 13 (Video S1). Three animals in the PTV-2-inoculated group developed mild incoordination of hind limbs starting at 15 DPI and partially recovered 4 days later. All animals in the PTV-11-inoculated group developed signs consistent with TE including hind limb incoordination, ataxia, posterior paresis, and quadriparesis (Video S2) starting at DPI 9. Mentation and ambulation scoring results are summarized in Table 4. A majority of virus-inoculated pigs (PTV-2: *n* = 5 and PTV-11: *n* = 7) developed a mild diarrhea beginning at DPI 7 (PTV-2) and DPI 8 (PTV-11).

### 3.3. Necropsy and Histopathologic Examination

**Figure 2:**
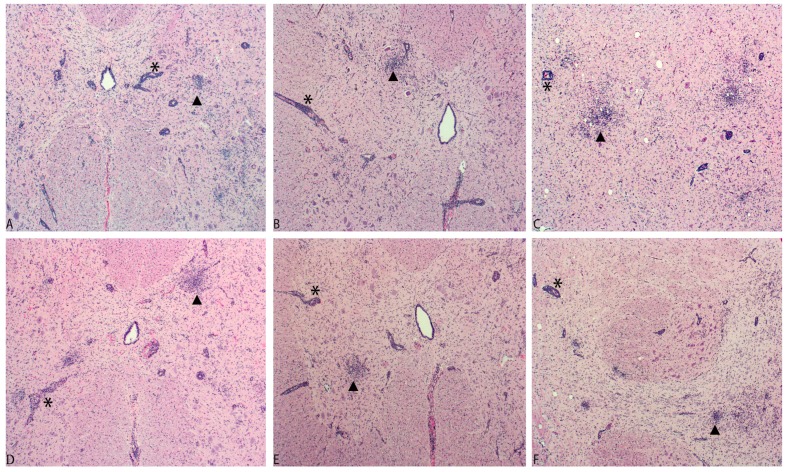
Pig No. 74, inoculated with PTV-2, day post-inoculation 11 (DPI 11). Lymphoplasmacytic myelitis with expansion of the Virchow–Robin spaces (*) and multifocal areas of gliosis (▲) in cervical spinal cord (**A**), lumbar spinal cord (**B**), and midbrain (**C**); Pig No. 77 inoculated with PTV-11, day post-inoculation 11 (DPI 11) Lymphoplasmacytic myelitis with expansion of the Virchow–Robin spaces (*) and multifocal areas of gliosis (▲) in cervical spinal cord (**D**), lumbar spinal cord (**E**), and midbrain (**F**).

Macroscopic examination of control and both infected groups was unremarkable. All animals in the PTV-2-inoculated and PTV-11-inoculated groups presented histological lesions in the spinal cord ganglia; cervical spinal cord, thoracic spinal cord, lumbar spinal cord; obex; pons; and midbrain (Figure 2 and Table 5). The severity of the histological lesions at different levels of the spinal cord were commonly higher in the PTV-11-inoculated group compared to PTV-2-inoculated group. Lesions were noted less commonly in the cerebellum, cerebrum at the level of the diencephalon, and frontal cortex in all virus-inoculated pigs (Figure 2 and Supplemental Figure S1).

### 3.4. Serum Neutralization Test

Serology results are summarized in Table 6. No antibodies against PTV-2 and PTV-11 were detected at DPI 0. Neutralizing antibodies were detected at DPI 11 in all virus-inoculated animals and remained detectable at 21 DPI.

### 3.5. Polymerase Chain Reaction

PCR results are summarized in Table 6. PTV RNA was not detected in feces or CNS tissue on negative control animals. PTV RNA was detected in feces and CNS tissue in all infected animals in each treatment group at time of necropsy.

### 3.6. Virus Re-Isolation

Cytopathic effect and positive IFA results were detected in all samples from virus-inoculated animals. PTV RNA was detected by PCR in all samples. Results are summarized in Table 7.

## 4. Discussion

The experimental inoculation of CDCD pigs in this study successfully demonstrated the neuropathogenicity of PTV-2 USA/IA65463/2014 and PTV-11 USA/IA09592/2013 isolates. A majority of pigs in the virus-inoculated groups developed mild hind limb incoordination to quadriparesis. The most severe clinical signs were noted in PTV-11-inoculated animals; however, all pigs in virus-inoculated groups developed histologic lesions consistent with TE.

Next generation sequencing of isolates PTV-2 USA/IA65463/2014 and PTV-11 USA/IA09592/2013 and subsequent phylogenetic and comparative sequence analyses confirmed these two field isolates as PTV-2 and PTV-11, respectively. PTV-2 USA/IA65463/2014 is genetically closely related to 3 European strains (AY392534, GQ293229, AY392533) isolated from CNS tissues of swine with CNS disorders, and to a Chinese strain (GU446660) isolated from CNS tissues of swine with enteric, respiratory, and CNS signs. PTV-11 USA/IA09592/2013 is genetically related to the PTV-11 prototype strain (AF296096) [16] and three other PTV-11 strains (AY392550, GQ293238, AY392536) isolated in Europe. Each strain was isolated from CNS tissue of pigs with CNS disorders, with the exception of GQ293238 whose background information was not available [28]. The inconsistencies shown in the phylogenetic trees for the VP1 capsid protein and 3D^pol^ genes when compared to the polyprotein gene tree agrees with previous report by Villanova et al. [29].

Although this study was not conceived to compare the pathogenicity of these strains, less severe clinical signs were observed in the PTV-2-inoculated animals than in PTV-11-inoculated animals. Intermittent ataxia was observed in three animals and ataxia was noted in one animal inoculated with PTV-2. All animals in the PTV-11-inoculated group developed clinical signs consistent with TE with three animals developing quadriparesis. Based on the number of animals that developed clinical signs consistent with TE and the severity of clinical signs and histologic lesions, it appears that PTV-2 USA/IA65463/2014 is less virulent than PTV-11 USA/IA09592/2013. However, the authors recognize that the small sample size used in this study and inoculation route limits the interpretation of the findings and/or extrapolation to field situations. It is interesting to note that despite the mild clinical signs noted in the PTV-2-inoculated group, histologic lesions were observed in all animals.

To our knowledge, this is the first experimental inoculation using a PTV-2 autochthonous U.S. strain after the initial description of PTV-2 strain o3b made by Long et al. in 1966 [30]. The distribution and severity of lesions are similar in both studies. Lesions are widely distributed in the CNS including the spinal cord, brain stem, and cerebellum, and to a lesser extent the cerebrum.

This is the first description of PTV-11 in the U.S. Originally, serotype PTV-11 was identified by Hahnefeld et al. as a PEV-1 strain in 1965 [31], and was further characterized as PTV-11 by Zell el al. in 2001 [16]. The comparative neuropathogenicity of European PTV-11 strains and this novel PTV-11 identified in the United States is not known.

Further work is warranted to assess the prevalence and geographic distribution of PTV serotypes in the U.S.

## Figures and Tables

**Table 1:** Experimental design.

Groups	Inoculum	Passage	No. of Animals	Route of Inoculation
Control	Cell culture media ^1^	-	4	Intravenous
PTV-2	10^6^ TCID_50_ PTV-2	8	7	Intravenous
PTV-11	10^6^ TCID_50_ PTV-11	7	7	Intravenous

^1^ Eagle’s minimum essential media. PTV-2: PTV-2 inoculated group, PTV-11: PTV-11 inoculated group. TCID_50_: median tissue culture infective dose.

**Table 2:** Mentation and ambulation scoring system.

Score	Mentation	Ambulation Score
0	Normal	Normal
1	Reduction in alertness	Mild incoordination of rear legs
2	Marked depression and head tilt	Intermittent ataxia of rear legs
3	2 plus seizures or opisthotonus	Anterior and/or posterior ataxia ± knuckling
4	3 plus events of seizures and opisthotonus	Posterior paresis, paralysis or quadriparesis

**Table 3:** Histopathologic severity score.

Score	No. of Cell Layers in Virchow-Robin Spaces	Areas of Gliosis
0	None	None
1	1	1–2 foci
2	2	3–4 foci
3	3	>4 foci
4	>3	-

**Table 4:** Clinical evaluation by animal.

Inoculum	Animal ID	Mentation Score	Ambulation Score ^1^	Ambulation Score by DPI ^2^											Diarrhea
				1	3	5	7	9	11	13	15	17	19	21	
PTV-2	61	**0**	**2**	0	0	0	0	0	0	0	1	2	2	0 *	+
	67	**0**	**0**	0	0	0	0	0	0 *	-	-	-	-	-	-
	68	**0**	**2**	0	0	0	0	0	0	0	1	2	1	0 *	-
	72	**0**	**3**	0	0	0	0	0	0	2	3 *	-	-	-	+
	73	**0**	**2**	0	0	0	0	0	0	0	2	2	0	0*	+
	74	**0**	**0**	0	0	0	0	0	0 *	-	-	-	-	-	+
	76	**0**	**0**	0	0	0	0	0	0	0	0	0	0	0 *	+
PTV-11	64	**0**	**2**	0	0	0	0	0	2	2	2	2	2	2 *	+
	65	**0**	**3**	0	0	0	0	0	3 *	-	-	-	-	-	+
	70	**0**	**4**	0	0	0	0	2	4 *	-	-	-	-	-	+
	71	**0**	**2**	0	0	0	0	2	0	0	0	0	0	0 *	+
	75	**0**	**4**	0	0	0	0	2	4 *	-	-	-	-	-	+
	77	**0**	**4**	0	0	0	0	2	4 *	-	-	-	-	-	+
	78	**0**	**3**	0	0	0	0	0	3	3	3	3	2	2 *	+

^1^ Highest ambulation score noted; ^2^ Recorded ambulation score by day post inoculation. * Last registered score for that animal. Plus sign (+) indicates animal with diarrhea, minus sign (-) indicates diarrhea not observed for that animal. PTV-2: PTV-2 inoculated group. PTV-11: PTV-11 inoculated group.

**Table 5:** Histopathologic severity score and distribution of lesions.

Location	Severity Score			Distribution	
	Control	PTV-2	PTV-11	PTV-2	PTV-11
Spinal ganglia	0	3	3	100%	100%
Cervical spinal cord	0	4	5	100%	100%
Thoracic spinal cord	0	3	5	100%	100%
Lumbar spinal cord	0	5	6	100%	100%
Sciatic nerve	0	0	0	0%	0%
Obex	0	4	6	100%	100%
Pons	0	4	5	100%	100%
Midbrain	0	4	5	100%	100%
Cerebrum plus diencephalon	0	2	4	86%	71%
Cerebellum	0	1	3	57%	71%
Front cortex	0	1	2	57%	71%

**Table 6:** Polimerase chain reaction (PCR) and serum virus neutralization test (SVN) results.

Inoculum	Animal No.	Necropsy DPI	PCR (CT) ^1^		SVN DPI ^2^		
			Feces	CNS	0	11	21
Cell Media ^3^	62	21	0	0	1/2	1/2	1/2
	63	21	0	0	1/2	1/2	1/2
	66	11	0	0	1/2	1/2	-
	69	11	0	0	1/2	1/2	-
PTV-2	61	21	26.16	33.26	1/2	1/16	1/32
	67	11	18.23	27.84	1/2	1/16	-
	68	21	26.24	31.69	1/2	1/32	1/16
	72	16	18.72	30.53	1/2	1/16	-
	73	21	22.62	32.46	1/2	1/16	1/32
	74	11	18.79	26.95	1/2	1/16	-
	76	21	24.78	28.67	1/2	1/16	1/16
PTV-11	64	21	21.75	31.97	1/2	1/64	1/64
	65	11	18.59	26.23	1/2	1/64	-
	70	11	17.78	24.43	1/2	1/32	-
	71	21	21.64	31.38	1/2	1/32	1/64
	75	11	18.57	24.62	1/2	1/128	-
	77	11	20.0	24.48	1/2	1/32	-
	78	21	21.92	32.01	1/2	1/16	1/32

^1^ Lowest cycle threshold (CT) value in CNS samples and feces collected at necropsy. ^2^ Serum virus neutralization assay at day post inoculation. ^3^ Eagle’s minimum essential media. Minus sign (-) indicates sample unavailable (animal euthanized prior collection date).

**Table 7:** Results of virus isolation attempts on CNS tissues from pigs inoculated with PTV-2 or PTV-11.

Inoculum	Animal ID	CPE P0	CPE P1	IFA P1	PCR ^1^
PTV-2	61	+	+	+	33.87
	67	+	+	+	18.36
	68	+	+	+	35.13
	72	+	+	+	31.52
	73	+	+	+	33.13
	74	+	+	+	22.11
	76	+	+	+	31.85
PTV-11	64	+	+	+	34.2
	65	+	+	+	28.78
	70	+	+	+	28.42
	71	+	+	+	21.57
	75	+	+	+	28.03
	77	+	+	+	28.14
	78	+	+	+	34.08

^1^ Lowest cycle threshold (CT) detected for that animal; CPE: Cytopathic effect; IFA: Indirect immunofluorescence; P0: Passage 0; P1: Passage 1; Plus sign (+) indicates positive result for that test.

## Supplementary Materials

The following are available online at www.mdpi.com/1999-4915/9/7/179/s1, Figure S1: Pig No. 72, inoculated with PTV-2 (DPI 11). Lymphoplasmacytic myelitis with expansion of the Virchow–Robin spaces (▲) and multifocal areas of gliosis (*) in the cervical spinal cord (**A**) and lymphoplasmacytic ganglioneuritis (**B**). Pig No. 70 inoculated with PTV-11 (DPI 11) Lymphoplasmacytic myelitis with expansion of the Virchow–Robin spaces (▲) and multifocal areas of gliosis (*) in the thoracic spinal cord (**C**) and lymphoplasmacytic ganglioneuritis (◄) in spinal ganglion (**D**). Video S1: Hind limb ataxia, PTV-2-inoculate, pig No. 72, day post-inoculation 16. Video S2: Paresis, PTV-11-inoculated, pig No. 70, day post-inoculation 10.
