# Supplementary figures and images for: Development of Polioencephalomyelitis in Cesarean-Derived Colostrum-Deprived Pigs Following Experimental Inoculation with Either Teschovirus A Serotype 2 or Serotype 11
Source: Viruses. 2017 Jul 8;9(7):179. doi: 10.3390/v9070179 (PMC5537671; doi:10.3390/v9070179)

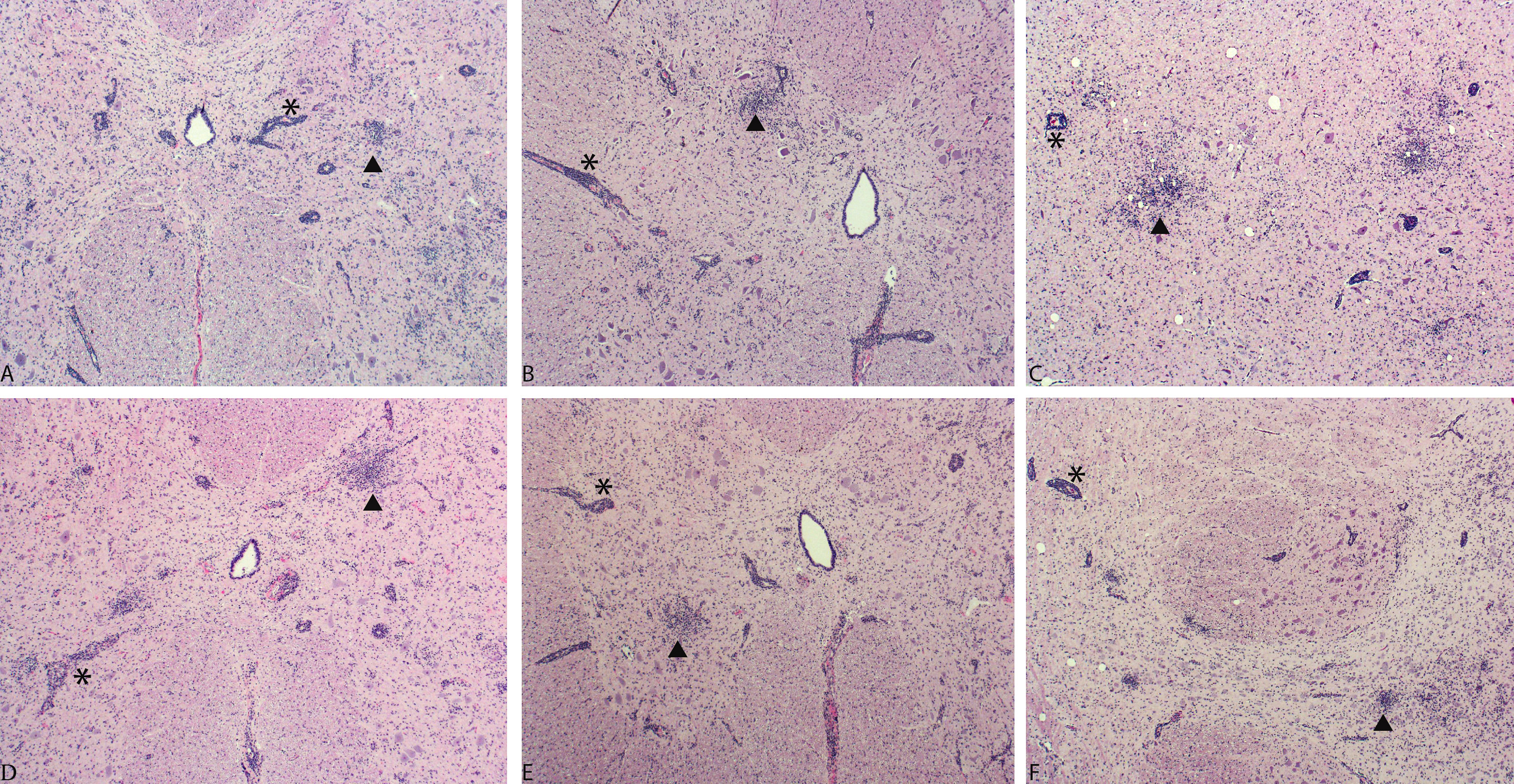

Supplement: Supplementary file 1 [file viruses-09-00179-s001.zip › viruses-199949-supplementary/Teschovirus Matias Ferreyra et al. Figure 2.png]

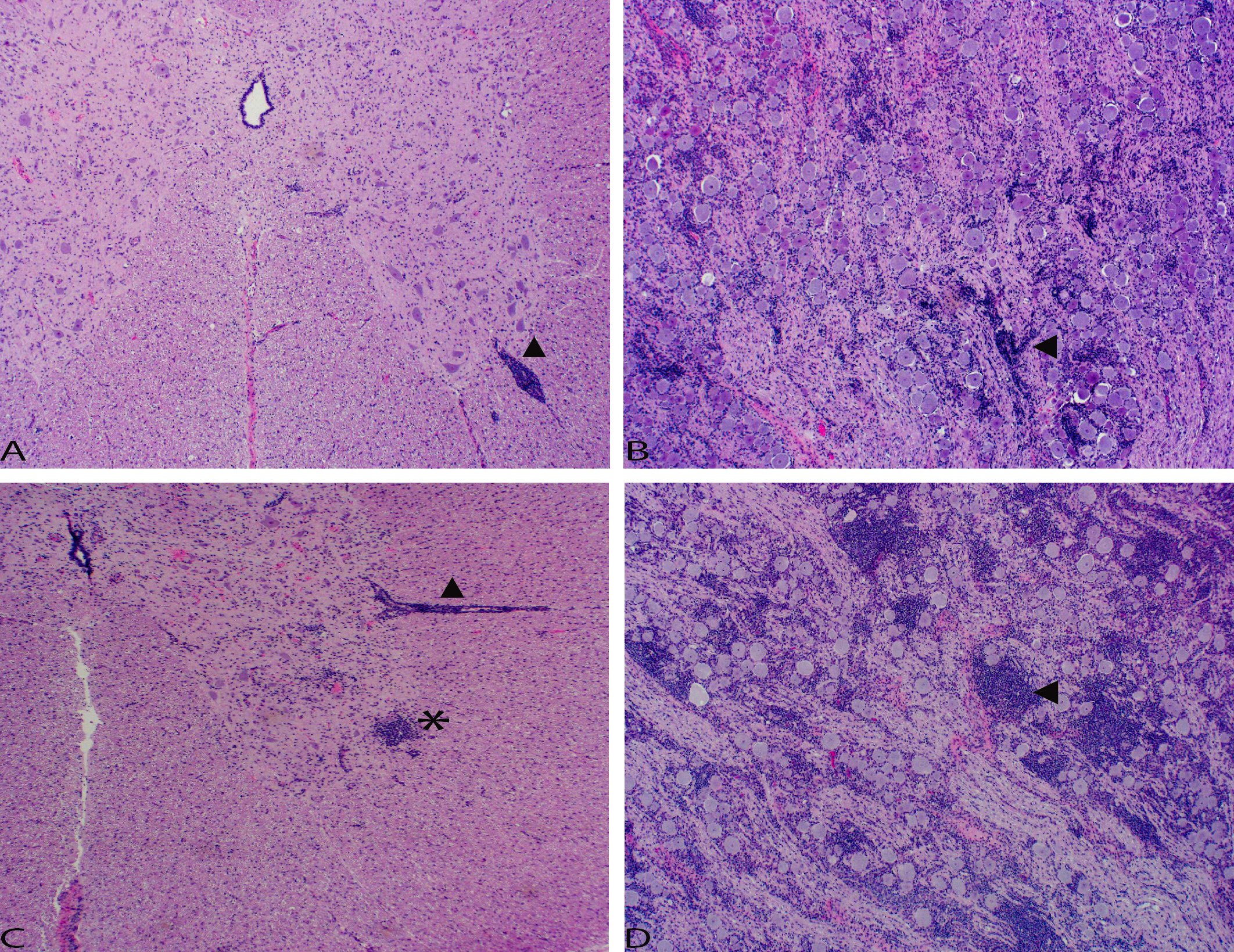

Supplement: Supplementary file 1 [file viruses-09-00179-s001.zip › viruses-199949-supplementary/Teschovirus Matias Ferreyra et al. Figure S1.png]
